# Cetuximab-decorated and NIR-activated Nanoparticles Based on Platinum(IV)-prodrug: Preparation, Characterization and *In-vitro* Anticancer Activity in Epidermoid Carcinoma Cells

**DOI:** 10.22037/ijpr.2020.113439.14303

**Published:** 2021

**Authors:** Yu Wang, Xin-Ming Zhang, Yu Sun, Hui-Lin Chen, Ling-Yun Zhou

**Affiliations:** a *School of Pharmacy, Wannan Medical College, Wuhu 241002, China. *; b *School of Chemistry and Materials Science, Anhui Normal University, Wuhu 241002, China. *; c *Institute of Synthesis and Application of Medical Materials, Wannan Medical College, Wuhu 241002, China.*

**Keywords:** Nanoparticles, Cetuximab, Targeted drug delivery, Near-infrared irradiation, Pt(IV)-prodrug

## Abstract

Platinum-based drugs are the mainstay of chemotherapy regimens in a clinic, but their use is seriously limited by severe side effects and drug resistance. A cetuximab-decorated drug delivery system can selectively deliver drugs into EGFR-highexpressing cancer cells to prevent the shortcomings of platinum-based chemotherapy. Here, cetuximab-decorated and near-infrared (NIR)-activated nanoparticles based on Pt(IV)-prodrug (abbreviated as Cetuximab-Pt-INPs) was constructed. First, PEGylated Pt(IV)-prodrug was synthesized by a condensation reaction between *c,c,t*-[Pt(NH_3_)_2_Cl_2_(OOCCH_2_CH_2_COOH)(OH)] and MPEG-PLA. Then, Pt(IV)-prodrug and indocyanine green co-encapsulated nanoparticles (Pt-INPs) were prepared through an ultrasonic emulsification method. Finally, Cetuximab-Pt-INPs were obtained by decorating Pt-INPs with cetuximab as a targeting vector. The optimized Cetuximab-Pt-INPs exhibited a spherical core-shell shape of 138.5 ± 0.96 nm. *In-vitro *cellular uptake and cytotoxicity assays revealed that more Cetuximab-Pt-INPs with NIR irradiation were selectively taken up by A431 cells, thereby leading to higher cytotoxicity. These multifunctional nanoparticles may have promising potential for targeted and effective therapy against EGFR-highexpressing cells of epidermoid carcinoma.

## Introduction

Ever since cisplatin (CDDP) had received FDA approval for cancer treatment in 1978, platinum-based anticancer drugs have become the mainstay of chemotherapy regimens in clinics. However, the more extensive use of them is limited, owing to severe side effects and drug resistance. In order to overcome these limitations, various drug-delivery systems have been developed based on diverse organic and inorganic materials (1, 2). Among these therapy strategies, selective drug delivery is an important approach with great potential for overcoming problems associated with the systemic toxicity of platinum-based chemotherapy. In particular, actively targeted nanoparticles demonstrate their remarkable selective targeting effect of delivering chemotherapeutics and augmenting therapeutic effectiveness (3, 4). Numerous ligands have been explored for such purposes, including aptamers, peptides, and carbohydrates, although antibodies (Ab) are perhaps the most frequently employed (5-7). Antibodies can be conjugated on the surface of NPs being able to specifically target antigens or receptors overexpressed in tumors (8). One of the most relevant mAb targets with a higher clinical relevance is the epidermal growth factor receptor (EGFR), which is often overexpressed in a variety of cancer cells (9, 10). Cetuximab, a widely used anti-EGFR antibody, consists of a chimeric mouse-human mAb directed against the extracellular domain of EGFR. After the surface of NPs was modified with cetuximab, NPs can bind and block the epidermal growth factor receptor (EGFR) with high affinity, show an enhanced antiproliferative activity over the same nanoparticles without the targeting moiety (9, 11-13).

Another promising strategy to overcome the shortcomings of platinum-based anticancer drugs is to use platinum(IV) complexes as prodrugs. Compared with square-planar Pt(II) complexes, Pt(IV) complexes always have an octahedral structure, which is quite inert toward ligand substitution and therefore avoid off-target reactions that deactivate (14-16). Additionally, the axial ligands play an important role in drug targeting and delivery, which can be used in a quite wide variety of chemical modifications facilitating the conjugation to the polymer. For example, the Pt(IV)-derivatives with succinic acid/s in the axial position/s are well suited for this purpose, because one carboxylic group is axially linked to the Pt core while the other is available for further reactions with the designed bio-vectors through amide or ester bonds (17-19).

Currently, combined treatment of near-infrared (NIR) thermotherapy and chemotherapy are also widely used to augment the cytotoxicity of chemotherapeutic agents. Indocyanine green (ICG) is an FDA-approved tricarbocyanine dye that can be absorbed and fluoresce in the region of 650-850 nm. In order to avoid the degradation and instability of ICG, a variety of nano-sized systems encapsulating this agent have been constructed. For example, ICG was encapsulated in various nanoparticles to overcome the drawbacks mentioned above and improve anticancer cytotoxicity. In addition, with the codelivery of ICG and anticancer drugs, the nano-sized carriers could achieve a combinative effect of chemotherapy and photothermotherapy (20-22). Thus, ICG-encapsulated NPs would be ideal because they cannot only remain the advantages of traditional nanoparticles but also have NIR-sensitivity and active targeting ability at the same time. Our group also has successfully prepared ICG-loaded nanoparticles for chem-photothermal therapy (23-25). Those nanoparticles with the assistance of NIR irradiation could carry heat and drug into cancer cells and obviously enhanced the chemo-photothermal therapeutic efficacy (22).

In this study, we aim to develop an optimized formulation for preparation of Pt(IV)-prodrug loaded and ICG-encapsulated targeting NPs (Cetuximab-Pt-INPs), where cetuximab was chosen as the targeting vector and ICG was chosen to enhance the the cytotoxicity of Pt(IV)-prodrug. Furthermore, the physicochemical properties, *in-vitro *cellular uptake behavior and antitumor activity of Cetuximab-Pt-INPs were investigated in epidermoid carcinoma cells.

## Experimental

*Materials*

Cisplatin was purchased from Shandong Boyuan Pharmaceutical CO., Ltd (Shandong, China). The anti-EGFR monoclonal antibody cetuximab was purchased from MedChemExpress (New Jersey, USA). The block copolymer Maleimide-Poly(ethylene glycol 3400)-block-Poly(L-lactic acid 34000) (Mal-PEG-PLA) and Methoxyl-Poly(ethylene glycol 2000)-block-Poly(L-lactic acid 8000) (MPEG-PLA) were purchased from Ponsure Biological CO., Ltd (Shanghai, China). Enhanced Cell Counting Kit-8 (CCK-8) and DAPI were purchased from Beyotime Biotechnology Co. Ltd. (Shanghai, China). Annexin V-FITC/PI apoptosis detection kit was purchased from BD Biosciences (New Jersey, USA). N,N’-diisopropylcarbodiimide(DIC), 4-dimethylaminopyridine (DMAP) and indocyanine green (ICG) were purchased from Sinopharm Chemical Reagent CO., Ltd (Shanghai, China). Traut’s reagent (2-iminothiolane) was obtained from Sigma-Aldrich (Shanghai, China). All solvents used in this study were analytic grade and used without further purification.

*Synthesis of PEGylated Pt(IV)-prodrug*

The overall synthesis of PEGylated Pt(IV)-prodrug (abbreviated as MPEG-PLA-Pt) involved three steps.

*Synthesis of c,c,t-[Pt(NH*_3_ *)*_2_ *Cl*_2_ *(OH)*_2_ *]*

The compound *c,c,t*-[Pt(NH_3_)_2_Cl_2_(OH)_2_] (complex 1) was synthesized according to a previously method (23, 26). Hydrogen peroxide (30 wt%, 60 mL) was added drop wise to a round bottom flask containing cisplatin (1.0 g, 3.33 mmol). The reaction mixture was heated to 75 °C for 5 h. The bright yellow solution was kept at room temperature in the dark for overnight to allow crystallization of the product. Yellow crystals were separated by filtration, washed with cold water, ethanol and diethyl ether, and vacuum dried. The product was obtained as bright yellow powder. Yield: 0.974 g (87.6%).

*Synthesis of c,c,t-[Pt(NH*_3_ *)*_2_ *Cl*_2_ *(OOCCH*_2_ *CH*_2_ *COOH)(OH)]*

The Pt(IV)-prodrug *c,c,t*-[Pt(NH_3_)_2_Cl_2_(OOCCH_2_CH_2_COOH)(OH)] (complex 2) was synthesized according to a procedure described in the literature (17, 27). Succinic anhydride (240 mg, 2.40 mmol) was added to a suspension of complex 1 (800 mg, 2.40 mmol) in DMSO (20 mL). The reaction mixture was stirred at room temperature in the dark for 24 h. DMSO was then removed under reduced pressure. The residue was dispersed in acetone and precipited a pale yellow solid. Cold diethyl ether was used to wash them several times. The product was dried in a vacuum for 48 h. Yield: 216 mg (83.1%).

*Synthesis of MPEG-PLA-Pt*

The PEGylated Pt(IV)-prodrug (complex 3) was synthesized by a condensation reaction between* c,c,t*-[Pt(NH_3_)_2_Cl_2_(OOCCH_2_CH_2_COOH)(OH)] and MPEG-PLA according to a literature procedure (28). Briefly, complex 2 (86.8 mg，20 μmol), DIC (46.5 mg，300 μmol) and DMAP (12.2 mg，100 μmol) were dissolved in DMSO (200 μL) and stirred at room temperature. After 30 min, the mixture was added to a DMSO solution (300 μL) of MPEG-PLA (200 mg). The resulting mixture was allowed to react at room temperature for 72 h with continuous stirring under N^2^ atmosphere. After that, cold deionized water (10 mL) was added to the reaction mixture. Then, an amicon ultra-15 centrifugal filter (10 K MWCO) was used to isolate unreacted prodrugs from aqueous suspension medium with ultracentrifuge (3500 *×g*, 15 °C, 3 min). The upper residue was washed twice by cold deionized water and followed by freeze-drying. The final product MPEG-PLA-Pt was obtained as a white solid (yield: 85.6%).

*Preparation of Pt(IV)-prodrug and ICG co-encapsulated nanoparticles*

The Pt(IV)-prodrug and ICG co-encapsulated nanoparticles (Pt-INPs) were prepared from MPEG-PLA-Pt, Mal-PEG-PLA and ICG by using a previously reported sonication method with some modification (29, 30). Briefly, amphiphilic copolymer (weight ratio of MPEG-PLA-Pt/Mal-PEG-PLA=1/1) was dissolved in dichlormethane (DCM) at concentrations of 1 mg/mL. ICG was dissolved in ultrapure water at concentrations of 1 mg/mL. To generate the Pt-INPs solution, 2 mL amphiphilic copolymer solution, 1 mL ICG solution and 7 mL ultrapure water were mixed together using a sonicator (Scientz-2D, China) at a frequency of 20 kHz and power of 285 W. After ultrasonic emulsified for 6 min, the resulting mixture was stirred at 300 rpm in dark for 24 h, allowing the slow evaporation of DCM and Pt-INPs solution formation.

To conjugate targeted ligands on the surface of Pt-INPs, cetuximab was thiolated for 1 h at room temperature by reacting them with a 20-fold excess of Traut’s reagent and 2 mM EDTA. Ellman’s reagent was used to determine the average number of sulfhydryl groups per thiolated cetuximab. To prepare cetuximab-decorated nanoparticles, a 1:100 molar ratio of thiolated cetuximab to maleimide moieties in polymeric mixed nanoparticles (Pt-INPs) was reacted in aqueous solution for 24 h at 4 °C. Free activated maleimide groups were blocked using 0.5 μL of 2-mercaptoethanol. Then an amicon ultra-15 centrifugal filter (10 K MWCO) was used to isolate fresh Cetuximab-Pt-INPs from aqueous suspension medium with ultracentrifuge (Cence-H1850R, China) at 4500 *×g* for 20 min.

*Characterization of Pt(IV)-prodrug and ICG co-encapsulated nanoparticles*

The formed Cetuximab-Pt-INPs were characterized for their structural morphologies. The particle size, polydispersity index, and zeta potential were evaluated by dynamic light scattering (DLS) (Brookhaven BI-200SMC, USA) at 25 °C, and the structural morphology was further verified using a transmission electron microscopy (TEM) (JEM-100XII, Japan) with negative stain method.

The encapsulation efficiency (EE) and loading efficiency (LE) of ICG or drug encapsulated in NPs were detected as follows: After the removal of free ICG and drug by centrifugation at 4500 *×g* for 20 min, 1 mL Cetuximab-Pt-INPs was added to 9 mL DMSO, and then the mixture was subjected to sonication to destroy the NPs. The concentration of ICG encapsulated in NPs was analyzed using UV/vis spectrometer (Shimadzu UV-2450, Japan) at 779 nm. The amount of Pt (IV)-prodrug loaded in NPs was tested by ICP-MS (HITACHI P-4010, Japan). The total amount of drug loaded onto the surface of the NPs was calculated in percentages as described in the following formula, and the final concentration of platinum (Pt) was obtained in μM by ICP-MS. The EE and LE are acquired through the following formula:

EE (%) = ((weight of loaded drug)/(weight of initially added drug)) × 100 

 Equation 1.

LE (%) = ((weight of loaded drug)/(total weight of NPs))× 100

Equation 2.

The protein concentration of Cetuximab in Cetuximab-Pt-INPs were determined by the colorimetric microBCA protein assay kit (Thermo Fisher 1510-05820, Finland) (13, 31). A sample collected from the supernatant of Pt-INPs without cetuximab was used as a control. The concentrations of cetuximab range from 10 μg/mL to 100 μg/mL were used for a standard curve.

*Cell culture*

The human epidermoid carcinoma cell line A431 and human colon cancer cell line HT-29 were purchased from the Cell Bank of Chinese Academy of Sciences (Shanghai, China) and cultured in DMEM supplemented with 10% (v/v) FBS, 100 U/mL penicillin and 0.1 mg/mL streptomycin at 37 °C, 5% CO_2_. The cells were trypsinized and passaged every 2-3 days and taken at the logarithmic growth phase for the experiment.

*Cellular uptake*

Cellular internalization and intracellular distribution of drug-loaded NPs *in-vitro *were observed using confocal laser scanning microscopy (CLSM) (Leica SP8, GER). Briefly, cells (5 × 10^4^ cells/well) were seeded into a confocal dish with 2 mL DMEM and incubated for 24 h. The original medium was replaced with 1.5 mL fresh culture medium with free ICG or Cetuximab-Pt-INPs (all containing 30 μg/mL of ICG), and incubated for 2.5 h at 37 °C. To remove the unbound NPs, the cells were washed twice with PBS and then fixed with 4% PFA for 20 min. The nuclear dye DAPI was used as a positive control to stain nuclei in the experiment. Finally, the fixed cells were washed three times with PBS and subsequently observed by CLSM. The FL was imaged at λ_EX_ 405 nm for nucleolus location and λ_EX_ 633 nm for ICG. The cells incubated with ICG and Cetuximab-Pt-INPs were first exposed to 1.6 W/cm^2^ 808 nm laser irradiation for 3 min, and incubated for another 2.5 h and further investigated the photothermal influence on cellular uptake.

*In-vitro cytotoxicity and photothermal therapy*

The cytotoxicity of various formulations (free CDDP and Cetuximab-Pt-INPs) was evaluated by CCK8 assay and the IC_50_ values were calculated using GraphPad Prism Software(32, 33) . Briefly, the A431 cells seeded in 96-well plates (5 × 10^3 ^cells/well) and incubated with DMEM for 24 h. The original medium was replaced with a fresh culture medium with various concentration CDDP or drug-loaded nanoparticles (containing same concentration of ICG and Pt). Cells cultured in DMEM medium containing 10% FBS (without exposure to drugs) were used as controls. To explore the photothermal therapeutic efficacy, the cells treated with Cetuximab-Pt-INPs were irradiated with NIR (1.6 W/cm^2^, 808 nm) for 1 min before incubation. While for chemotherapy alone, the cells were not exposed to NIR laser. After another 24 or 48 h incubation, a 10 μL enhanced cell counting kit-8 (CCK8) solution was immediately added to each well and homogeneously mixed. After incubated for another 3 h, the absorbance of each well at a wavelength of 450 nm was recorded by a microplate reader (Thermo Fisher). The cell viability of the samples was calculated as follows:

 Cell viability (%) = ((A_test_ – A_blank_)/(A_control _- A_blank_)) × 100 

Equation 3.

Where A_test_ and A_control_ represent the intensity determined for cells treated with different samples and for control cells, respectively, and A_blank_ is the absorbance of the wells without cells.

*Cell apoptosis*

The cell apoptosis analysis was evaluated by using the annexin V-FITC apoptosis detection kit. Briefly, the A431 cells were seeded into 6-well plates (2 × 10^5^ cells/well) with 2 mL DMEM and incubated for 24 h. Then the original medium was replaced with a fresh culture medium with various concentrations of CDDP or Cetuximab-Pt-INPs (containing 40 μg/mL ICG and 50 μM Pt) and subsequently irradiated with a 1.6 W/cm^2^ 808 nm laser for 3 min. Cells cultured in DMEM medium without drugs were used as controls. While for chemotherapy alone, the cells treated with Cetuximab-Pt-INPs were not exposed to NIR laser. After another 12 or 24 h incubation, the cells were then rinsed, resuspended in 195 μL binding buffer and incubated for 10 min with 5 μL annexin V-FITC at room temperature, followed by adding 10 μL PI. The solutions were gently mixed and analyzed with the FACSVerse flow cytometry from Becton Dickinson (New York, USA).

## Results and Discussion

*Syntheses and characterization of PEGylated Pt(IV)-prodrug*

**Figure 1:**
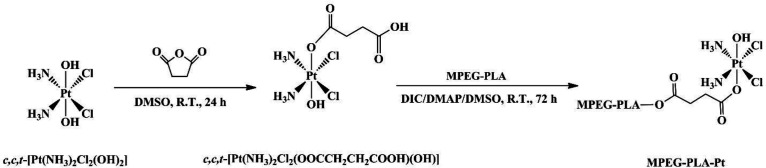
Synthetic route of PEGylated Pt(IV)-prodrug (MPEG-PLA-Pt).

**Figure 2:**
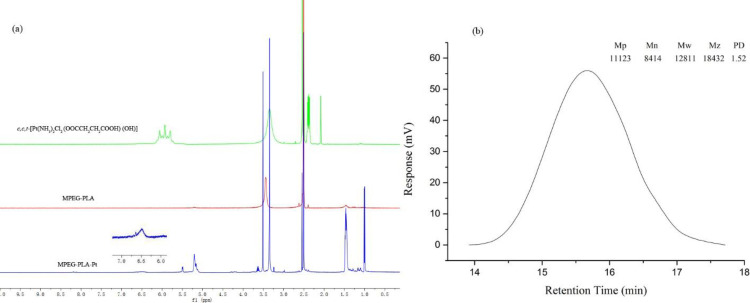
Characterization of MPEG-PLA-Pt. (a) ^1^H-NMR spectra. (b) GPC spectra

PEGylation has been widely applied in drug delivery systems for the modification of proteins, peptides, and vehicles. PEGylated nanoparticles can prolong bloodstream circulation time by averting opsonization and uptake by the mononuclear phagocyte system and consequently accumulate in tumor tissue rather than normal tissues through the enhanced permeability and retention (EPR) effect (34). In this work, the PEGylation of cisplatin was performed by conjugating with MPEG-PLA. The detailed synthetic route of PEGylated Pt(IV)-prodrug (MPEG-PLA-Pt) was shown in Figure 1. First, cisplatin was oxidized with H_2_O_2_ into complex 1, which consisted of two hydroxyl groups at its axial positions. One of the two hydroxyl groups was then reacted with succinic anhydride to form complex 2. Finally, the free carboxyl group was reacted with the OH groups of MPEG-PLA to form complex 3 using the DIC/DMAP method. The chemical structure was investigated by ^1^H-NMR and ESI-MS. The ^1^H-NMR (DMSO-*d*_6_) spectrum of *c,c,t*-[Pt(NH_3_)_2_Cl_2_(OOCCH_2_CH_2_COOH)(OH)] (Figure 2a) illustrated characteristic peaks of -NH_3_ at 5.93 ppm and -CH_2_- at 2.39 ppm(18). ESI-MS (m/z): 432.8 (M^+^, Calcd 433.1). After it was reacted with MPEG-PLA to form complex 3, the chemical shift of -NH_3_ moved to 6.49 ppm and a new chemical shift of -CH_2_- of succinic anhydride residue appeared at 2.51 ppm, supporting the structure from Complex 2. The ^1^H-NMR (DMSO-*d6*) spectrum of MPEG-PLA-Pt also illustrated characteristic peaks of PEG chain at 3.53 ppm. The peaks of 5.22 ppm and 3.37 ppm respectively pertained to -COCH(CH_3_)O- and -COCH(CH_3_)O- of PLA segment (35). The peaks of 1.45 ppm belonged to methyl group of -OCH_3_. Furthermore, Figure 2b illustrates the chromatogram and molecular weight data resulting from the GPC analysis of MPEG-PLA-Pt after synthesis. The curve was unimodal and symmetrical. The molecular weight [MW] and polydispersity was 12.8 kDa and 1.52, respectively.

*Preparation and Characterization of Cetuximab-Pt-INPs*

**Figure 3:**
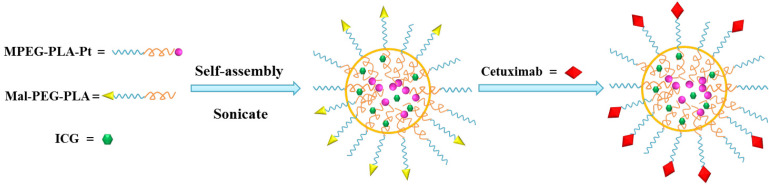
Scheme for the preparation of Cetuximab-Pt-INPs

**Figure 4:**
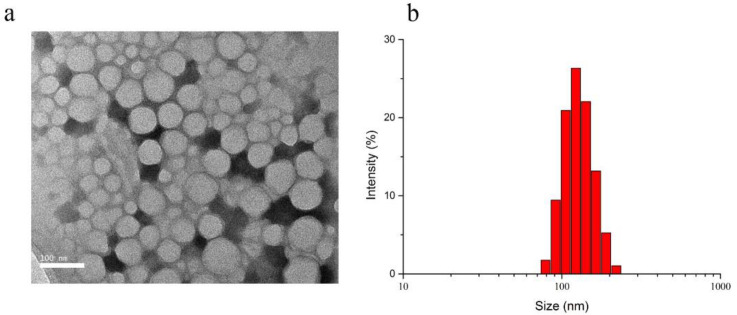
Characterization of Cetuximab-Pt-INPs. (a) Typical TEM image of Cetuximab-Pt-INPs. Scale bars represent 100 nm. (b) Size distribution of Cetuximab-Pt-INPs measured by DLS

The scheme for Cetuximab-Pt-INPs preparation is presented in Figure 3. In order to construct the cetuximab-modified and NIR-activited drug delivery system, the Pt(IV)-prodrug loaded NPs (abbreviated as Pt-INPs) were first self-assembled from MPEG-PLA-Pt, Mal-PEG-PLA and ICG through a single-step sonication method. And then the resulting mixture was gently stirred in dark to evaporate organic solvent. After that, Pt-INPs were further functionalized with the specific targeting ligand cetuximab through the reaction between the maleimide group of nanoparticles and sulfydryl group of cetuximab, resulting in an expected drug delivery system. TEM and DLS were used to evaluate the morphological characterization of Cetuximab-Pt-INPs. As shown in Figure 4, TEM confirmed that Cetuximab-Pt-INPs had a spherical core-shell shape with an average diameter in the range of 78 to 255 nm. DLS showed the hydrodynamic sizes of Cetuximab-Pt-INPs were 138.5 ± 0.96 nm, which was in close proximity with the particle size obtained from TEM measurement. The surface charge diameter and size polydispersity index of Cetuximab-Pt-INPs was found to be -12.53 ± 0.61 mv and 0.05 ± 0.03, respectively, which inferred the good stability and midrange polydispersity.

Encapsulation efficiency (EE) and loading efficiency (LE) are important criteria for the evaluation of NPs. Therefore, ICG and MPEG-PLA-Pt were encapsulated into the NPs via sonication method and the non-encapsulated free molecules were removed by centrifugation and filtration. Based on the Equation 1, EE of ICG and MPEG-PLA-Pt in Cetuximab-Pt-INPs were 20.85 ± 1.04% and 40.62 ± 3.67%, respectively, while LE of ICG and MPEG-PLA-Pt in Cetuximab-Pt-INPs were approximately 6.98 ± 0.22% and 6.75 ± 1.08%, respectively, calculated based on the Equation 2. These results indicated that Cetuximab-Pt-INPs had a compact structure by the aggregation effect of ICG in the core of the nanoparticles.

To examine the binding affinity, the Cetuximab-Pt-INPs were analyzed by BCA analysis. The purple color and OD value of Cetuximab-Pt-INPs were determined by a spectrophotometer. Based on the standard curve, there was about 55.89% (163 μg) of cetuximab added was incorporated on the surface of Cetuximab-Pt-INPs. Our data are in agreement with those reported in the literature by other authors (31, 36).

*In-vitro cellular uptake of Cetuximab-Pt-INPs*

**Figure 5:**
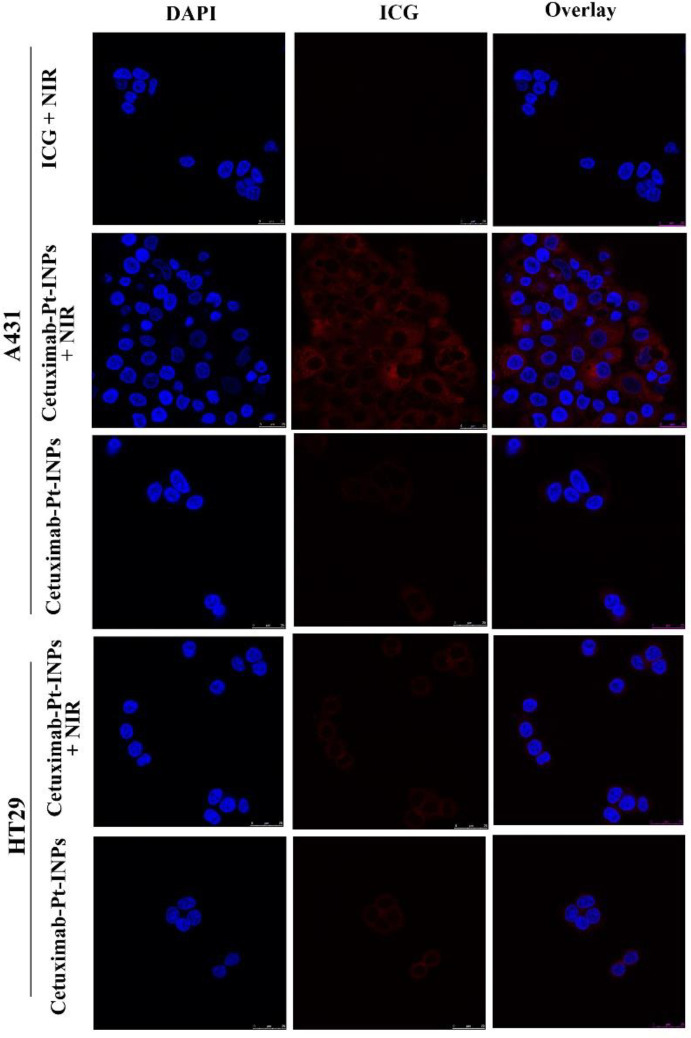
Cellular uptake of A431 cells and HT-29 cells treated with free ICG, Cetuximab-Pt-INPs (with or without NIR laser irradiation) observed by CLSM. Nuclei (blue) were stained with DAPI; red represented the fluorescence of ICG. Scale bars represent 25 μm

It is demonstrated that cetuximab can induce the EGFR-mediated endocytosis via dynamin-dependent and dynamin-independent pathways in different cell types (29, 37).In order to test whether cetuximab increased the uptake and accumulation of Cetuximab-Pt-INPs, human epidermoid carcinoma cell line A431 was used as the EGFR-highexpressing cell line, and human colon cancer cell line HT-29 was used as the EGFR-lowexpressing cell line, and then the uptake of Cetuximab-Pt-INPs and free ICG was measured by CLSM (Figure 5) (38, 39). A high level of ICG fluorescence was observed in A431 cells when treated with Cetuximab-Pt-INPs, which indicated that a great number of Cetuximab-Pt-INPs had entered into the cytoplasm of A431 cells. On the contrary, almost no ICG fluorescence was observed in A431 cells treated with free ICG. Upon NIR irradiation at 808 nm, the fluorescence intensity of ICG was further strengthened when A431 cells were incubated with Cetuximab-Pt-INPs. For HT-29 cells, weak ICG fluorescence was observed because of the low expression of EGFR by these cells. These results indicated that the modification of cetuximab on the surface of nanoparticles resulted in efficient and specific association with EGFR-highexpressing cells. The encapsulation and irradiation of ICG promoted the uptake of Cetuximab-Pt-INPs for A431 cells. The raised cellular uptake of Cetuximab-Pt-INPs may contribute to improving the chemotherapeutic and phototherapeutic efficiency. This competitive uptake inhibition supports a specific EGFR receptor-mediated endocytosis mechanism of cetuximab INPs, as already described for different cetuximab NPs (12, 39 and 40).

*In-vitro cytotoxicity and photothermal therapy of Cetuximab-Pt-INPs*

**Figure 6:**
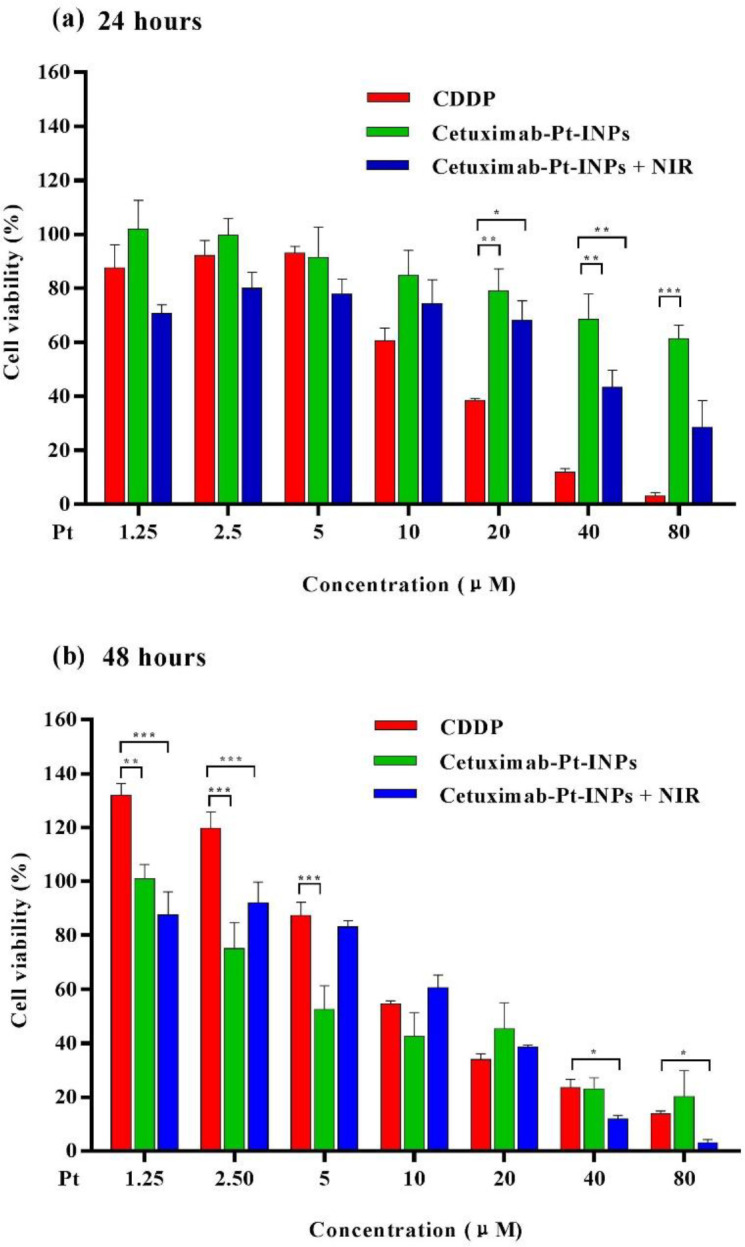
*In-vitro* cellular viability of A431 cells after 24 h or 48 h incubation with CDDP, Cetuximab-Pt-INPs (with or without NIR laser irradiation). ^*^p < 0.05, ^**^p < 0.01, ^***^p < 0.001. Data were presented as mean ± SD. (n = 3)

In order to demonstrate the* in-vitro *therapeutic efficacy, cytotoxicity of different Pt-prodrug loaded formulations against A431 cells were quantitatively evaluated by CCK8 assay. As shown in Figure 6, Cetuximab-Pt-INPs (with or without NIR laser irradiation) and free CDDP inhibited tumor cells proliferation in a time- and dose-dependent manner, confirming that higher drug concentration and longer incubation time are essential for the drug to effectively kill tumor cells. During the first 24 h of incubation, about 68.51% of cells kept alive after being incubated with Cetuximab-Pt-INPs at the Pt concentration of 40 μM, while about 43.44% of cells remained viable under the same treatment conditions upon NIR irradiation (1.6 W/cm^2^, 808nm, 1 min) during the drug incubation. Both cell viabilities were higher than that of free CDDP, which may occur due to the slower and sustained release of Pt(IV)-prodrug from NPs. After 48 h incubation, the cell viability of Cetuximab-Pt-INPs at the Pt concentration of 40 μM decreased to 23.03%, which was close to that of CDDP but still higher than 11.97% of Cetuximab-Pt-INPs upon NIR irradiation. It was worth noting that Cetuximab-Pt-INPs with NIR irradiation showed pronounced cell-killing ability at high Pt concentration (10 and 80 μg/mL) due to remarkable hyperthermia of photothermal effect at relatively high ICG concentrations (23, 41). These results demonstrate that NIR laser irradiation is an effective way to enhance the therapeutic effect of Cetuximab-Pt-INPs for cancer treatment via the photothermally enhanced drug release rate and cellular internalization of Cetuximab-Pt-INPs. Based on the cell viability data, the IC_50_ values of free CDDP and Pt(IV)-prodrug loaded nanoparticles were estimated. After 24 h incubation, the IC_50_ value of Cetuximab-Pt-INPs with or without NIR irradiation was 39.55 ± 1.39 μM and 127.9 ± 1.33 μM, respectively. They were higher than 14.16 ± 1.06 μM of CDDP. After 48 h incubation, with the further degradation of platinum(IV)-prodrug, IC_50_ values of Cetuximab-Pt-INPs with or without NIR irradiation decreased to 13.32 ± 1.63 μM and 10.04 ± 1.17 μM, respectively. They were similar to 14.40 ± 1.19 μM of CDDP. These results demonstrate that the encapsulation of ICG and NIR irradiation could effectively reduce A431 cells viability, which was in accordance with the results of cellular uptake. Thus, Cetuximab-Pt-INPs with NIR activation had photothermal-chemotherapy therapy.

*Cell apoptosis of Cetuximab-Pt-INPs*

**Figure 7:**
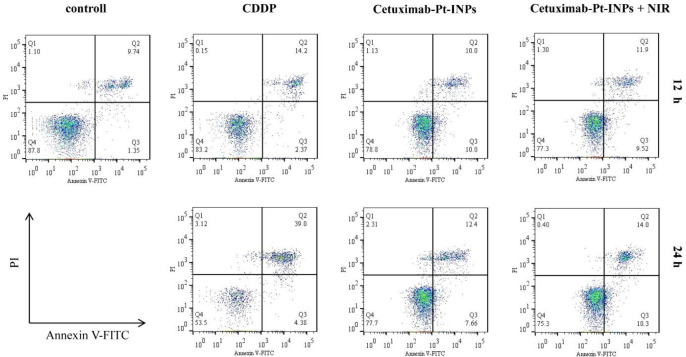
Flow cytometry analysis of A431 cells after 12 h or 24 h incubation with CDDP, Cetuximab-Pt-INPs (with or without NIR laser irradiation).

The annexin V-FITC/PI double staining assay was performed to determine whether the growth inhibitory effect of the cetuximab-modified nanoparticles was associated with cell apoptosis. Positive PI and Annexin V-FITC cells were defined as late apoptosis/necrotic cells. The percentages of cell apoptosis are depicted in Figure 7. Upon NIR irradiation and after 12 h incubation, about 21.42% of A431 cells were induced apoptosis by Cetuximab-Pt-INPs, which was a little higher than that of Cetuximab-Pt-INPs without NIR irradiation or free CDDP. Meanwhile, the increasing apoptosis of these drugs were observed over time. Cetuximab-Pt-INPs with NIR irradiation caused about 24.30% apoptosis rate after 24 h incubation. It was still higher than that of Cetuximab-Pt-INPs without NIR irradiation but lower than that of CDDP. These results indicated that the cytotoxic effect of Cetuximab-Pt-INPs on A431 cells could be improved by the laser irradiation. It was worth noting that this influence was a little weaker than our previous similar studies, which may due to the shorter irradiation time (23, 24) . However, it can still be concluded that Cetuximab-Pt-INPs with NIR activation had photothermal-chemotherapy therapy.

## Conclusion

We successfully developed a novel kind of cetuximab-decorated and NIR-activated nanoparticles that can be selectively internalized into cancer cells via receptor-mediated endocytosis. The Cetuximab-Pt-INPs were first prepared with MPEG-PLA-Pt, Mal-PEG-PLA, and ICG through the ultrasonic emulsification method and then modified with cetuximab on their surface. The optimized Cetuximab-Pt-INPs exhibited a spherical core-shell shape of 138.5 ± 0.96 nm. Due to the cetuximab-decorated surface, Cetuximab-Pt-INPs can selectively deliver drugs to EGFR-highexpressing A431 cells. Upon NIR irradiation, the Cetuximab-Pt-INPs show enhanced anti-tumor efficacy by increasing cellular uptake and cell apoptosis. Therefore, these findings demonstrated the possibility of targeted delivery of PEGylated Pt(IV)-drugs and their enhanced *in-vitro *therapeutic effect to human epidermoid carcinoma cells.
